# Introduction to thematic series “new sociological perspectives on inequality”

**DOI:** 10.1186/s40711-021-00145-y

**Published:** 2021-02-17

**Authors:** Mike Savage, Chunling Li

**Affiliations:** 1 London School of Economics and Political Science, London, UK; 2 Institute of Sociology, Chinese Academy of Social Sciences, Beijing, China

Over the past decade, the social scientific analysis of inequality has been renewed with a vigour and urgency which is desperately necessary to respond to the burgeoning challenges of the twenty-first century. The tempered optimism of the last decades of the twentieth century based on economic and technological growth which appeared to have played a transformative role in improving the lives of millions of poor people over much of the world has given way to a much bleaker sense of the intractability of emerging challenges of the twenty-first century. Climate change, intensifying medical crises most obviously manifest in the Covid-19 pandemic, and deepening militarist and nationalist politics have become increasingly evident. Many of these concerns crystallise around a recognition of the persistence, and even escalation of inequalities across many dimensions of economic and social life since the late twentieth century. Only 20 years ago, inequality had been seen to be a marginal and even receding issue which would be sidelined by modernisation and economic growth. Some sociologists (Pakulski and Waters 1996) had diagnosed “the end of class”. Influential sociologist Manuel Castells (1996) had even proclaimed “the end of patriarchalism”. Although claims that racial divisions were being alleviated in an allegedly “post-racial” environment were generally treated with scepticism, they were still widely aired, especially with the election of Barack Obama as President of the United States. How dated and parochial this optimism now seems in the grim year of 2020, when the Covid-19 pandemic exposed the festering, brutal reality of inequalities across numerous dimensions all too visibly.

A major intellectual driver of the renewed social scientific interest in inequality has been the development of a powerful branch of the discipline of economics which conducted trailblazing long-term analysis of inequality trends within nations and across the globe. This was a movement of seismic importance since previously, the study of inequality (or “distribution”) was a backwater of the economics discipline. From the 1970s, an inspiring group of economists including Anthony Atkinson, Amartya Sen and Joseph Stiglitz insisted on the need to take inequality seriously and developed new measurement tools for studying inequality trends. By the early twenty-first century, these crystallised in the development of the World Top Incomes database in 2011, and in 2015, renamed as the World Wealth and Income Database. In the past decade, a number of pioneering publications from the group associated with the “World Inequality Lab” have attracted much interest and dramatically re-energised the study of inequality (Piketty 2014, 2020; Atkinson 2015; Zucman 2015; Alvaredo et al. 2018, see special issues of *British Journal of Sociology*, BJS 2015). A central intellectual move has been to shift attention away from the analysis of poverty towards the entire distribution with a special concentration “top end” incomes and wealth, thereby placing the most significant beneficiaries of economic growth under the spotlight in a more critical and forensic light than was previously the case. This readily mapped onto increasing public concerns about the burgeoning fortunes of the super-rich amidst the stagnation of opportunities for many.

In the past decade, this important current of research has fused with an awareness of the need to analyse inequality globally. Studies of inequality have therefore moved away from the North American and European heartlands to embrace a more global perspective (compare Piketty and Saez’s 2003 initial study of the United States with Piketty 2014 and Piketty 2020). This current research partly emerges from the critique of national framings of inequality, as if national boundaries were the inevitable receptacle for social divisions, rather than being part of wider global dynamics (see Bhambra 2007; Anand and Segal 2015; Milanovic 2016; Savage 2021b). This sensitivity also recognises the distinctiveness of the trajectories of developing countries with more serious inequality problems and the need to bring these nations more closely into view. In developing nations, inequality has always been the core theme of social scientists, especially sociologists. However, they have experienced the same scenario of older optimism transitioning into a later grim mood a few decades later, just as European and American social scientists have, but in an even more sped-up fashion. Even in the first decade of the twenty-first century, major emerging economies, such as China, Russia, Brazil, India and South Africa, were being hailed as success stories in a period of rapid economic growth. Social scientists saw the rise of the middle class and the reduction of poverty as being of major historical significance (Kharas 2010; Li 2010; Li et al. 2013; Li 2015; Bartelt and Sievers 2017). There were also situations where long-term rising inequality in China, Russia and Brazil seemed to be alleviated. Sociologists claimed that the emergence of the black middle class in South Africa and the Dalit middle class in India were breaking through older forms of social segregation (Southall 2004; Still 2014). Social scientists complacently predicted that a "middle-class society" was on the rise.

However, the financial crisis in 2008 ended the golden age of high economic growth in these countries and also tarnished their dreams of the “middle-class society” as a realistic aspiration for all citizens (Madheswaran and Vani 2020). Slowing and fluctuating economies led to greater concerns about rising inequality and made debates about emerging class divisions more prominent. The “New Rich”, who had benefitted much more from economic growth, had made an astonishing fortune, but it became increasingly apparent that this was more likely to lead to wealth polarisation and social conflict in these societies which continued to have a huge poor population and less social welfare provision. When European and American social scientists renewed their interest in inequality since 2000, they therefore joined forces with social scientists from many major emerging economies who also came to have a stronger sense of the urgency needed to deal with inequality. The experience of escalating income and wealth inequality in these nations explains why these topics became a new focus of inequality research. Sociologists in these developing countries, alongside Western colleagues, are beginning to move their attention to these topics from traditional themes of class and social stratification.

One of the most important features of this new area of inequality research is the emphasis on the need for inter-disciplinary collaboration (notably, Piketty 2014, Savage 2014). This demonstrates how inequality is not purely an economic phenomenon but has wider social, cultural and political ramifications. This call explicitly opens up the possibility of dialogue between economics and sociology, aspects of which have already taken place, for instance, through special issues of *The British Journal of Sociology* in 2015 and 2021 (BJS 2015, e.g. see Savage 2021a, Savage 2021b). Our special issue further responds to this opportunity by elaborating sociological perspectives which build on and engage with the approaches also developed by economists. Our papers use new and innovative empirical studies from different nations including Brazil, China, Russia, South Korea and the UK. The papers in this special issue, versions of which were first presented at a conference organised by the Chinese Academy of Social Sciences in Kunming in 2019, therefore all indicate how sociologists can contribute to the renewal of the study of inequality, drawing on their own theoretical and methodological repertoires.

We would like to highlight three major features of these contributions which we hope will attract a wide inter-disciplinary readership.

Firstly, as we explain above, many of the papers in this issue take up the need to sociologically make case studies from the Global South more central. Here, we are taking a lead from the work of economists such as Milanovic (2016) and Anand and Segal (2015) who have emphasised the need for a global analysis of inequality which de-centres the significance of Europe and North America and more accurately represents the variegated picture from different parts of the globe (see also Simson and Savage 2020; Savage 2021b; Li et al. 2013). Sociological class analysis needs to follow suit. It has previously been dominated by perspectives from the Global North, especially Europe and North America, and it is a vital task to redress this imbalance and bring a greater variety of global experiences into the picture. We thus have papers from China (Li and Fan), Russia (Mareeva), Brazil (Scalon et al.) and South Korea (Shin), as well as a comparative study of developed nations (Chauvel et al.) and the UK (Savage and Meersohn Schmidt).

Secondly, in developing this more global perspective, it is vital to reflect on the sociological significance of income and wealth distributions for conceptualising stratification and inequality. Previously, within sociology, there has been a tendency for sociologists to downplay these aspects, partly out of deference to economists, and partly to highlight the significance of categorical divides which economists have been slower to emphasise (e.g. Goldthorpe 2010). Notably, class, gender, race and ethnicity were frequently conceptualised as analytically independent from income and wealth distributions, even though it was recognised that they correlated in crucial ways. Even the study of social class, which intuitively might be expected to map onto economic distributions, was held apart from it in the influential work of British sociologist John Goldthorpe, who emphasised that class was rooted in employment and occupation and should not be conflated with levels of income (Goldthorpe 2000, 2010).

By contrast, the papers in this issue consider how income and wealth inequalities are themselves bound up with social inequality dynamics, and thus seek a much wider engagement with recent work in economics. This is in large part due to the need to pay attention to the dramatic economic changes across the Global South and their significance for shaping social inequality and the analysis of social stratification. A major theme is to consider how class analysis itself might be changed by a focus on income (and wealth) dynamics. Scalon et al. (2021) in the Brazilian context, Mareeva (2020) in Russia and Shin (2020) in South Korea consider how a close analysis of the income distribution modifies our perception of class in these two nations, notably by drawing attention to increased polarisation. This work can build on arguments within class analysis which emphasise the need to de-couple class analysis from studies of the “occupational order” (whilst recognising that occupations are still significant carriers of resources) by placing them within a more multidimensional perspective. In fact, there is a burgeoning interest in conceptualising class in terms of “capitals, assets and resource” (Savage et al. 2005) which offer a theoretical hook to bring the analysis of wealth squarely into a class analysis. The Great British Class Survey (Savage et al. 2013, 2014, 2015) has been an influential model of how alternative approaches to class might be developed which can bring in the distribution of economic capital into view (see also Jodhka et al. 2017).

Finally, and most specifically, many of our papers take up the need to bring the analysis of wealth inequality fully into our sociological analysis. The study of wealth inequality is much less developed than for income, and this is one of the major areas that Piketty (2014, 2020) and Zucman (2015), Piketty and Zucman (2014) have emphasised. This is an issue where sociological research has much to offer, since wealth inequality intersects with numerous categorical axes and intensifies racial and gender divides (e.g. Shapiro 2017 on race, Mears 2015 and Glucksberg 2018 on gender). Nonetheless, it is challenging to empirically take forward this agenda, partly due to the difficulty of measuring wealth accurately, especially recognising the way that it is divided into separate components (e.g. housing assets, savings, financial investments, pensions). The papers here represent important statements which seek to advance our understanding of these issues and which seek to use a focus on wealth inequality as a means of renewing class analysis more broadly in a period in which the “asset economy” (see Adkins et al. 2020) is of pivotal importance. Chauvel and his co-authors (2021) thus reflect on how the distribution of wealth towards top percentiles changes our picture of the middle class through showing how wealth tends to be more polarised than income and therefore indicates that there is not a large class with middling amounts of wealth. Mareeva (2020) brings out, in the Russian case, how the pulling away of the very wealthy is somewhat at odds with the trends towards equalisation in the middle layers. Shin (2020) also demonstrates how wealth inequality does not map onto income inequality and thus needs special consideration. Li and Fan (2020) demonstrate the burgeoning importance of wealth inequality linked to the dynamics of housing markets in the Chinese context.

An important aspect of recognising the significance of wealth inequality is the way that its accumulation takes place over time, often long time periods. Wealth thereby requires us to understand temporal dynamics over a long time period, moving away from static cross-sectional analyses of social relationships. This therefore means that they overlap and intersect with people’s life histories and trajectories, meaning that social and personal identities intertwine. This point is underscored by Savage and Meersohn Schmidt (2020) who contribute to this broad endeavour by showing how the political attitudes and identities of precarious Britons need to be conceptualised in terms of their life-long experiences. They also show how taking this issue seriously also challenges conventional models of class consciousness, such as might be derived from occupational class models. Just as wealth is held in households and communities as well as on an individual basis, this also means that kinship dynamics are also implicated in processes of class formation. These points are also relevant to the studies of Li and Fan, and Shin regarding housing markets and wealth.

We therefore hope that these papers will be of wide interest to social scientists grappling with the changing dynamics of inequality across the globe, and hope they inspire further comparative research in this vein. A focus on burgeoning wealth inequality as a major driver of social divisions opens up a major agenda for a reinvigorated class analysis that can work in different national settings. This special issue offers some important insights which will assist this research agenda.

## References

[CR1] Adkins, L., M. Cooper, and M. Konings. 2020. *The asset economy*. London: Wiley.

[CR2] Alvaredo, F., L. Chancel, T. Piketty, E. Saez, and G. Zucman, eds. 2018. *World inequality report 2018*. Boston: Belknap Press.

[CR3] Anand, S., and P. Segal. 2015. The global distribution of income. In *Handbook of income distribution*, vol. 2, 937–979. London: Elsevier.

[CR4] Atkinson, A.B. 2015. *Inequality: what can be done?.* Boston: Harvard University Press.

[CR5] Bartelt, D.D., and A.A. Sievers. 2017. *The new middle class in India and Brazil: Green perspectives*. New Delhi: Academic Foundation.

[CR6] Bhambra, G. (2007). Rethinking modernity: Postcolonialism and the sociological imagination. Springer.

[CR7] BJS. 2015. Special issue on Piketty’s Capital in the 21^st^ century. *British Journal of Sociology* 65: 4.

[CR8] Castells, M. (1996). The rise of the network society, Oxford Blackwells.

[CR9] Chauvel, L., E. Bar Haim, A. Hartung, et al. 2021. Rewealthization in twenty-first century Western countries: the defining trend of the socioeconomic squeeze of the middle class. *Journal Chinese Sociology* 8: 4. 10.1186/s40711-020-00135-6. 10.1186/s40711-020-00135-6 PMC7797273 35822199

[CR10] Glucksberg, L. 2018. A gendered ethnography of elites: women, inequality, and social reproduction. *Focaal* 2018 (81): 16–28.

[CR11] Goldthorpe, J.H. 2000. *On sociology: numbers, narratives, and the integration of research and theory*. Oxford University Press.

[CR12] Goldthorpe, J.H. 2010. Analysing social inequality: a critique of two recent contributions from economics and epidemiology. *European Sociological Review* 26 (6): 731–744.

[CR13] Jodhka, S.S., B. Rehbein, and J. Souza. 2017. *Inequality in capitalist societies*. London: Taylor & Francis.

[CR14] Kharas, H. 2010. The emerging middle class in developing countries. In *OECD Development Centre working papers (285)*.

[CR15] Li, C. 2010. *China’s emerging middle class: beyond economic transformation*. New York: Brookings Institution Press.

[CR16] Li, C. 2015. *The rising middle classes in China*. Beijing: Social Sciences Academic Press & Paths International Ltd..

[CR17] Li, C., and Y. Fan. 2020. Housing wealth inequality in urban China: the transition from welfare allocation to market differentiation. *Journal Chinese Sociology* 7: 16.

[CR18] Li, P., M.K. Gorshkov, C. Scalon, and K.L. Sharma. 2013. *Handbook on Social Stratification in the Bric Countries: Change and Perspective*. New Jersey: World Scientific.

[CR19] Madheswaran, S., and B.P. Vani. 2020. *The middle class in world society: negotiations, diversities and lived experiences*. London: Routledge.

[CR20] Mareeva, S. 2020. Socio-economic inequalities in modern Russia and their perception by the population. *Journal Chinese Sociology* 7: 10 10.1186/s40711-020-00124-9. 10.1186/s40711-020-00124-9 PMC7327491 38624369

[CR21] Mears, A. 2015. Working for Free in the VIP: Relational work and the production of consent. *American Sociological Review* 80 (6): 1099–1122.

[CR22] Milanovic, B. 2016. *Global inequality: a new approach for the age of globalization*. Boston: Harvard University Press.

[CR23] Pakulski, J., and M. Waters. 1996. *The death of class*. London: Sage.

[CR24] Piketty, T. 2014. *Capital in the 21*^*st*^*century*. Boston: Harvard University Press.

[CR25] Piketty, T. 2020. *Capital and ideology*. Boston: Harvard University Press.

[CR26] Piketty, T., and E. Saez. 2003. Income inequality in the United States, 1913–1998. *The Quarterly Journal of Economics* 118 (1): 1–41.

[CR27] Piketty, T., and G. Zucman. 2014. Capital is back: wealth-income ratios in rich countries 1700–2010. *The Quarterly Journal of Economics* 129 (3): 1255–1310.

[CR28] Savage, M. 2014. Piketty’s challenge for sociology. *The British Journal of Sociology* 65 (4): 591–606. 25516340 10.1111/1468-4446.12106

[CR29] Savage, M. (2021a). The Return of Inequality: Social Change and the Weight of History. Harvard, MA: Harvard University Press.

[CR30] Savage, M. 2021b. *The return of inequality: social change and the weight of the past*. Boston: Harvard University Press.

[CR31] Savage, M., N. Cunningham, F. Devine, S. Friedman, D. Laurison, L. Mckenzie, A. Miles, H. Snee, and P. Wakeling 2015. *Social class in the 21*^*st*^*century*. London: Penguin.

[CR32] Savage, M., F. Devine, N. Cunningham, S. Friedman, D. Laurison, A. Miles, H. Snee, and M. Taylor. 2014. On social class, Anno 2014. *Sociology* 49 (6): 1011–1030.

[CR33] Savage, M., F. Devine, N. Cunningham, M. Taylor, Y. Li, J. Hjelbrekke, B. Le Roux, A. Miles, and S. Friedman. 2013. A new model of social class? Findings from the BBC’s Great British Class Survey Experiment. *Sociology* 47 (2): 219–250.

[CR34] Savage, M., and C. Meersohn Schmidt. 2020. The politics of the excluded: abjection and reconciliation amongst the British precariat. *Journal Chinese Sociology* 7: 23 10.1186/s40711-020-00134-7.

[CR35] Savage, M., A. Warde, and F. Devine. 2005. Capitals, assets, and resources: some critical issues. *The British Journal of Sociology* 56 (1): 31–47. 15777461 10.1111/j.1468-4446.2005.00045.x

[CR36] Scalon, C., A.J. Caetano, H. Chaves, et al. 2021. Back to the past: gains and losses in Brazilian society. *Journal Chinese Sociology* 8: 3 10.1186/s40711-020-00132-9.

[CR37] Shapiro, T.M. 2017. *Toxic inequality: how America’s wealth gap destroys mobility, deepens the racial divide, and threatens our future*. New York: Basic Books.

[CR38] Shin, K.Y. 2020. A new approach to social inequality: inequality of income and wealth in South Korea. *Journal Chinese Sociology* 7: 17 10.1186/s40711-020-00126-7.

[CR39] Simson, R., and M. Savage. 2020. The global significance of national inequality decline. *Third World Quarterly* 41 (1): 20–41.

[CR40] Southall, R. 2004. Democratic change and the black middle class in South Africa. *Canadian Journal of African Studies* 38 (3): 521–542.

[CR41] Still, C. 2014. *Dalits in neoliberal India: mobility or marganilization?* New Delhi: Routledge.

[CR42] Zucman, G. 2015. *The hidden wealth of nations: the scourge of tax havens*. Chicago: University of Chicago Press.

